# Aetiology and prognosis of community-acquired pneumonia at the Adult University Teaching Hospital in Zambia

**DOI:** 10.1371/journal.pone.0271449

**Published:** 2022-07-15

**Authors:** L. M. Ziko, T. W. Hoffman, S. Fwoloshi, D. Chanda, Y. M. Nampungwe, D. Patel, H. Bobat, A. Moonga, L. Chirwa, L. Hachaambwa, K. J. Mateyo

**Affiliations:** 1 Department of Internal Medicine, Pulmonary/Infectious Diseases Unit, University Teaching Hospital, Lusaka, Zambia; 2 The University of Zambia, School of Medicine, Lusaka, Zambia; 3 Adult Infectious Disease Centre, University Teaching Hospital, Lusaka, Zambia; 4 Department of Pulmonology, St. Antonius Hospital, Nieuwegein, The Netherlands; 5 University of Lusaka, Lusaka, Zambia; 6 Institute of Research and Development, Lusaka, Zambia; 7 Department of Infectious Diseases, University of Maryland, Baltimore, Maryland, United States of America; George Washington University, UNITED STATES

## Abstract

**Background:**

Community-acquired pneumonia (CAP) is a frequent cause of death worldwide, and in sub-Saharan Africa particularly. Human immunodeficiency virus infection (HIV) and tuberculosis (TB) influence pathogen distribution in patients with CAP. Previous studies in sub-Saharan Africa have shown different frequencies of respiratory pathogens and antibiotic susceptibility compared to studies outside Africa. This study aimed to investigate the aetiology, presentation, and treatment outcomes of community-acquired pneumonia in adults at the University Teaching Hospital in Lusaka, Zambia.

**Materials and methods:**

Three-hundred-and-twenty-seven patients were enrolled at the University Teaching Hospital in Lusaka between March 2018 and December 2018. Clinical characteristics and laboratory data were collected. Sputum samples were tested by microscopy, other TB diagnostics, and bacterial cultures.

**Results:**

The commonest presenting complaint was cough (96%), followed by chest pain (60.6%), fever (59.3%), and breathlessness (58.4%). The most common finding on auscultation of the lungs was chest crackles (51.7%). Seventy percent of the study participants had complaints lasting at least a week before enrolment. The prevalence of HIV was 71%. Sputum samples were tested for 286 patients. The diagnostic yield was 59%. The most common isolate was *Mycobacterium tuberculosis* (20%), followed by *Candida species* (18%), *Klebsiella pneumoniae* (12%), and *Pseudomonas aeruginosa* (7%). *Streptococcus pneumoniae* was isolated in only four patients. There were no statistically significant differences between the rates of specific pathogens identified in HIV-infected patients compared with the HIV-uninfected. Thirty-day mortality was 30%. Patients with TB had higher 30-day mortality than patients without TB (*p* = 0.047).

**Conclusion:**

*Mycobacterium tuberculosis* was the most common cause of CAP isolated in adults at the University Teaching Hospital in Lusaka, Zambia. Gram-negative organisms were frequently isolated. A high mortality rate was observed, as 30% of the followed-up study population had died after 30 days.

## Introduction

Community-acquired pneumonia (CAP) causes substantial morbidity and mortality in developing countries, particularly sub-Saharan Africa [1]. CAP can be caused by various types of pathogens, such as bacteria, fungi, viruses, and mycobacteria. Most studies on pneumonia aetiology have been performed in developed countries, and treatment algorithms have mainly been derived from and evaluated in this population [2]. In sub-Saharan Africa, CAP aetiology is considerably influenced by Human Immunodeficiency Virus (HIV) and tuberculosis (TB) co-infection [3].

The aetiology of CAP is further influenced by region, variable population characteristics, and seasonal variations. In a study done in Nigeria, *Streptococcus pneumoniae* was the most frequent pathogen in sputum from adult patients hospitalized for CAP. Other relatively common pathogens were *Klebsiella pneumoniae*, *Staphylococcus aureus*, and *Streptococcus pyogenes*. The susceptibility of the pathogens to antibiotics most frequently prescribed for empiric therapy, such as penicillins and cephalosporins, was low. The low antibiotic susceptibility was associated with higher mortality, as well as longer hospital stay in survivors [4]. Studies in Kenya also demonstrated that *Streptococcus pneumoniae* was the most prevalent pathogen, whereas *Influenza A* virus was the most common viral pathogen [5–7]. A recent study from a teaching hospital in Malawi found *Mycobacterium tuberculosis* (MTB) and *Streptococcus pneumoniae* to be the most common pathogens [8].

In patients infected with HIV, *Mycobacterium tuberculosis* is a common cause of pneumonia, as found in a study from Zambia [9]. Nyamande *et al*. showed similar results in South Africa, where *Mycobacterium tuberculosis* was the most common pathogen in HIV-infected patients, followed by *Streptococcus pneumoniae* [10]. *Pneumocystis jirovecii* had a low prevalence of 4.4% in the Zambian study, in contrast to a study done in Kenya showing a high prevalence of 37.2% in HIV patients from an urban hospital [11]. A study in Cameroon however, showed no difference in the aetiology of pneumonia and clinical outcomes between HIV infected and non-HIV infected patients [12].

Overall, few studies have been done on pneumonia in Zambia. Therefore, there are insufficient data to influence local treatment algorithms. This study aimed to investigate the aetiology, presentation, and treatment outcomes of community-acquired pneumonia in adults at the University Teaching Hospital in Lusaka, Zambia.

## Materials and methods

This was a prospective cohort study. Patients were enrolled at the University Teaching Hospital (UTH) in Lusaka, Zambia between March 2018 and December 2018. UTH is a tertiary referral center for patients from the whole of Zambia but also serves a local population from Lusaka, the capital city. Patients are either walk-in or referred from other facilities within Lusaka and other parts of the country. Participants were consecutively recruited from the medical admissions ward, inpatient wards and the medical outpatient department. Patients enrolled were aged 18 years and above, and met the World Health Organization definition of pneumonia for use in resource-limited settings [13]: cough with two or more of: fever/night sweats, tachypnoea, or chest pain. Alternatively, the diagnosis was made when any of the aforementioned criteria was present in combination with a chest X-ray suggestive of pneumonia. Patients with a strong alternative diagnosis such as pulmonary embolism or pulmonary oedema were excluded from the study, as were patients that developed a pneumonia after being admitted to the hospital for >48 hours or while on a ventilator. There were no other exclusion criteria. All patients provided written informed consent before enrolment. Ethical approval was obtained from the University of Zambia Biomedical Research Ethics Committee (UNZABREC) under reference number 005-09-17.

### Data collection

The investigators did not provide any direct clinical care to the participants. Data was collected by the researchers or a research assistant using a standardized form. Patient history was obtained after enrolment, when patients were in the admission ward or on the medical wards. Collected information included demographics, pulmonary symptoms, medication use, and HIV status. Risk factors for pulmonary disease based on local hospital data were collected including history of diabetes mellitus (clinical diagnosis), heart failure (clinical diagnosis), chronic kidney disease (diagnosed in accordance with KDIGO definition), [14] asthma (clinical diagnosis), stroke (clinical diagnosis), chronic obstructive pulmonary disease (clinical diagnosis), and hypertension (clinical diagnosis). Patients’ vitals including blood pressure, respiratory rate, pulse rate, and pulse oximetry were obtained, and a full physical examination was done. Peripheral venous blood was collected for full blood count (FBC), renal and liver function tests. For participants with unknown HIV status, Alere Determine^®^ and SD-Bioline^®^ serology tests for confirmation were offered in line with the Zambian National HIV testing algorithm, and this had no further impact on data collection [15]. CD4 counts were measured in anti-retroviral treatment-naïve patients with HIV, and also in patients with HIV who were already receiving treatment but for whom no CD4 count was on record in the preceding six months. HIV viral load was tested in patients with HIV who had received treatment for at least six months but did not have a documented viral load in the past 12 months.

Participants were asked to submit three sputum samples for analysis. Those unable to spontaneously expectorate were referred for bronchoscopy and bronchial lavage. Respiratory samples were sent to the laboratory for gram, Toluidine blue O, and Ziehl-Neelsen stains that were produced in-house. Respiratory specimens were also cultured on blood and chocolate agar. The VITEK 2^®^ COMPACT-platform (bioMérieux, St. Louis, MO) was used for identification and antimicrobial sensitivity testing. For patients with TB, Rifampicin resistance was determined by the Xpert MTB/RIF on theGeneXpert (Cepheid, Sunnyvale, CA). Patients or their next of kin were contacted telephonically after 30 days of their admission to determine mortality. Participants were under the care of attending physicians.

### Data analyses

There was no predefined sample size, as this study was intended as an exploratory analysis. Data analyses were performed using Statistical Package for Social Sciences (SPSS) for Windows version 24 (IBM Corporation, Armonk, NY). To compare continuous variables which were found to be normally distributed, using the Shapiro-Wilk test of normality, Student’s T-test was used and means and standard deviations (SD) were reported. For continuous variables that were found not to be normally distributed, the Mann-Whitney U test was used and medians and interquartile ranges were reported. Chi-squared and Fisher’s exact tests were used to quantify the association between categorical dependent variables and categorical independent variables. For all statistical tests, a p-value of less than 0.05 was considered significant.

## Results

**Fig 1:**
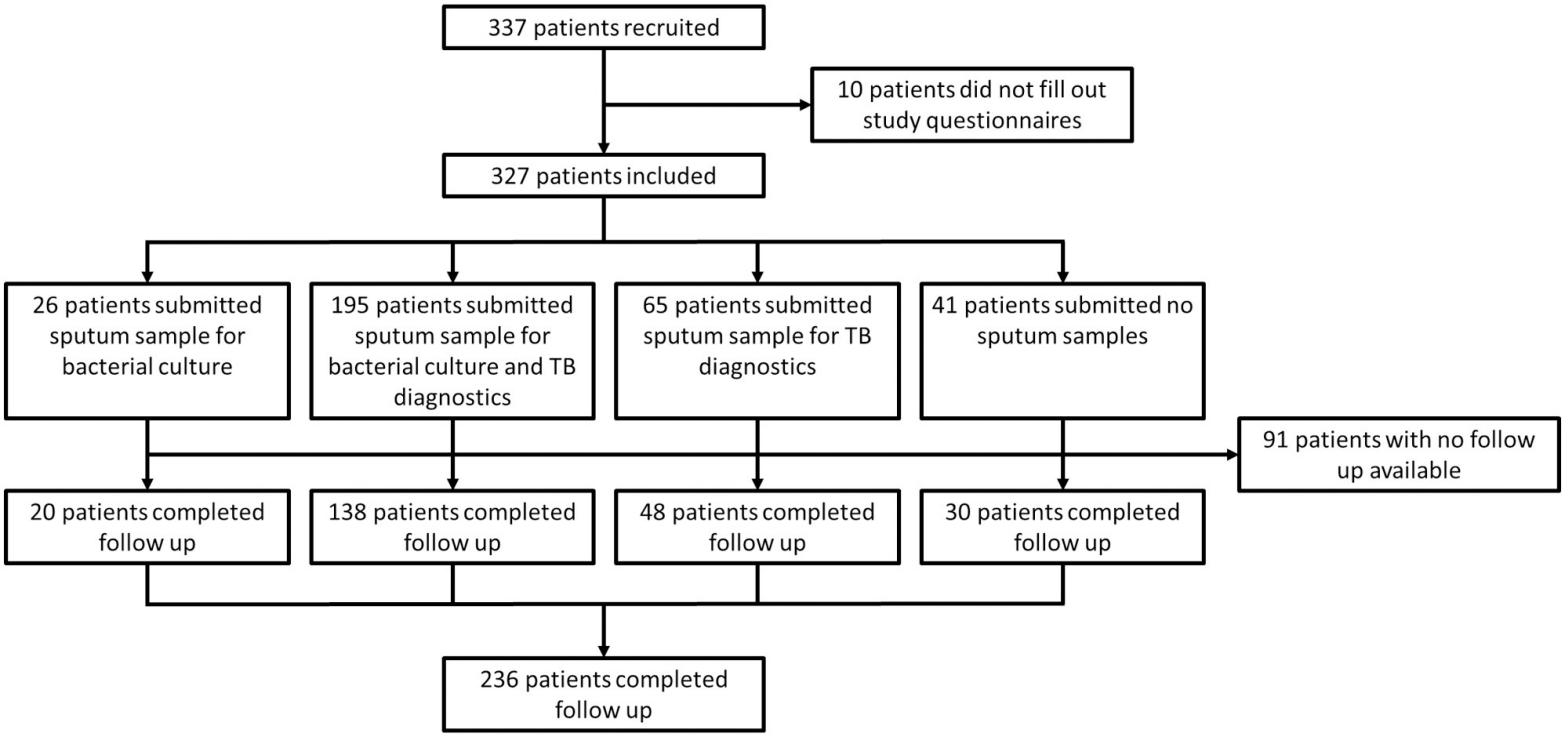
Study flow chart.

A total of 337 patients were recruited. 10 patients could not complete filling in of the study questionnaires. Of the 327 patients that were enrolled, for 41 no sputum samples could be collected. A total of 236 patients or their next of kin were contacted 30 days after enrolment to establish 30-day mortality. The study flow chart is shown in Fig 1.

Baseline characteristics are presented for the 327 included patients in Table 1. Forty-nine percent of the cohort was female (n = 161). The mean age was 41.2 years (SD± 16.3) years and 93% were in-patients (n = 305). Fifty-five percent of the study participants consumed alcohol (n = 179), and 33% smoked cigarettes (n = 107). Seventy-one percent of the participants with available HIV status (n = 296) was HIV positive (n = 209) and of these, 83% were on antiretroviral therapy (n = 173). HIV-negative patients were significantly older compared to HIV-positive patients (median age 38 years (IQR 27–54) versus 40 years (IQR 30–48), *p* = 0.004 (Mann-Whitney U test)), and a significantly larger proportion of HIV-negative patients was 65 years or older (30% versus 3%, *p* = 0.0001). Forty percent of all patients reported having previously had TB (n = 132). Other comorbid conditions included asthma and chronic obstructive pulmonary disease in 12 (4%) and 22 (7%) patients, respectively. Comorbidities were more often seen in HIV-negative patients, and this difference was statistically significant for TB, heart failure, and diabetes mellitus (Table 1). Cigarette smoking was also significantly more common in HIV-negative patients (44% versus 28%, *p* = 0.008).

**Table 1:** Baseline characteristics, also noted separately for patients who were HIV infected and HIV uninfected.

Characteristic	Total (n = 327)	HIV-positive (n = 209)	HIV-negative (n = 87)	*P*-value
**Demographics**				
Age in years, median (IQR)	39 (28–50)	40 (30–48)	38 (27–54)	0.004
Age > 65 years (%)	38 (11.6)	7 (3.3)	26 (29.9)	0.0001
Female (%)	161 (49.2)	105 (50)	39 (44.8)	0.40
Inpatients (%)	305 (93.3)	201 (96.2)	78 (89.7)	0.06
Intensive Care Unit (%)	1 (0.3%)	0 (0)	1 (1.1)	0.29
**Past medical history**				
Alcohol use (%)	179 (54.7)	116 (55.5)	48 (55.2)	0.93
Cigarette smoking (%)	107 (32.7)	58 (27.8)	38 (43.7)	0.0083
Tuberculosis (%)	132 (40.4)	104 (49.8)	22 (25.3)	0.00014
Renal failure (%)#	2 (0.6)	1 (0.5)	1 (1.1)	0.50
Heart failure (%)	19 (5.8)	6 (2.9)	11 (12.6)	0.0020
Chronic obstructive pulmonary disease (%)	22 (6.7)	13 (6.2)	8 (9.2)	0.33
Asthma (%)	12 (3.7)	5 (2.4)	5 (5.7)	0.17
Diabetes mellitus (%)	8 (2.4)	3 (1.4)	5 (5.7)	0.05
Cerebrovascular accident (%)	6 (1.8)	3 (1.4)	2 (2.3)	0.63
Self-reported antiretroviral therapy (%)	173 (52.9)	173 (82.8)	0 (0)	-
**Received antibiotics in previous month (%)**	218 (66.7)	160 (76.6)	45 (51.7)	<0.0001
Anti-tuberculous treatment	98 (30)	71 (34)	22 (25)	0.19
Trimethoprim/sulfamethoxazole prophylaxis	86 (26)	83 (40)	0 (0)	<0.0001
Other antibiotics	101 (31)	61 (29)	31 (36)	0.09
**Symptoms**				
Cough (%)	314 (96.0)	202 (96.7)	82 (94.3)	0.35
Breathlessness (%)	191 (58.4)	122 (58.4)	55 (63.2)	0.44
Chest pain (%)	198 (60.6)	120 (57.4)	60 (69.0)	0.070
Fever (%)	194 (59.3)	123 (58.9)	46 (52.9)	0.34
Haemoptysis (%)	64 (19.6)	27 (12.9)	31 (35.6)	<0.0001
Duration of Symptoms >1 week (%)	228 (69.7)	155 (74.2)	57 (65.5)	0.17
**Physical findings**				
Crackles (%)	169 (51.7)	115 (55.0)	44 (50.6)	0.43
Reduced air entry (%)	43 (13.1)	30 (14.4)	11 (12.6)	0.68
Normal auscultatory findings (%)	107 (32.7)	62 (29.7)	29 (33.3)	0.57
Oxygen use (%)	37 (11.3)	30 (14.4)	7 (8.0)	0.13
Oxygen saturation ≤ 90% (%)	98 (29.8)	65 (31.1)	24 (27.6)	0.34
Systolic blood pressure ≤ 90 mmHg (%)	45 (13.7)	36 (17.2)	6 (6.9)	0.03
Diastolic blood pressure ≤ 60 mmHg (%)	69 (21.0)	48 (23.0)	16 (18.4)	0.44
Temperature ≤ 35°C or ≥ 39.9°C (%)	0 (0)	0 (0)	0 (0)	1.00
Respiratory rate ≥ 30/min (%)	98 (29.8)	67 (32.1)	20 (23.0)	0.12
Body Mass Index, mean (SD)	20.3 (5.3)	19.7 (5.1)	21.4 (5.8)	0.025

SD, Standard Deviation, IQR Inter Quartile Range

^#^ patients meeting the KDIGO classification for chronic kidney disease [14].

The most common presenting complaint was cough (96%) followed by chest pain (60.6%), fever (59.3%), and breathlessness (58.4%). Haemoptysis was less common (19.6%), but was seen significantly more often in HIV-negative patients (36% versus 13%, *p*<0.0001). The most common finding on auscultation of the lungs was chest crackles (51.7%). Seventy percent of the study participants had complaints lasting at least a week before enrolment. Two hundred eighty-four patients (86%) had received antibiotics at the time of enrolment and the commonest antibiotics received were anti-tuberculous treatment (30%) and trimethoprim/sulfamethoxazole (26%; used as prophylaxis for *pneumocystis jirovecii* pneumonia in almost all cases). Other commonly prescribed antibiotics were cephalosporins (14%) and penicillins (13%).

The mean haemoglobin was 10.14g/dl (SD±3.34) whereas median urea was 5.71mg/dL (IQR 3.81–10) and serum creatinine 70.7μmol/L (IQR 53.5–101.5). The CD4 count was measured in 89 patients, of whom 64 patients had a CD4 count below 200 cells/μL (72%). Of these 64 patients, 7 were not using antiretroviral therapy (11%). The viral load was measured in 33 patients and was above 1000 copies/ml in 15 patients (46%). The median CD4 count and viral load were 96.5 cells/μL (IQR 25–220) and 311 copies/ml (IQR 20–73500) respectively in the HIV-infected participants.

Of the 327 study participants, 221 had sputum samples sent for bacterial culture, and 260 had samples sent for Toluideine Blue O and Ziehl-Neelsen staining and Xpert MTB/RIF. In 286 patients, at least one sputum sample was sent for either of the aforementioned diagnostics. In total, potentially pathogenic microorganisms were isolated in 170, giving a diagnostic yield of 59% in patients for whom sputum samples were available, and 52% for the whole cohort (Table 2). *Mycobacterium tuberculosis* was detected in 49 patients, of whom nine had rifampicin resistance. The second most frequent pathogen was *Candida species* (found in 39 patients). Other commonly identified microorganisms were *Klebsiella pneumoniae* (found in 27 patients), *Pseudomonas aeruginosa* (found in 16 patients), *Pseudomonas alcaligenes*, and *Acinetobacter baumanni* (both found in 11 patients). *Streptococcus pneumoniae* was identified in only four patients whereas *Pneumocystis jirovecii* was not found in any patient. Pathogens were significantly more often isolated in HIV-infected patients (66% versus 42%; *p* = 0.0007). There were no statistically significant differences between the types of pathogens isolated in HIV-infected patients compared with HIV uninfected patients.

**Table 2:** Potential pathogens identified from sputum samples in patients with pneumonia.

Microorganism	Total number	In HIV infected patients	In HIV uninfected patients	*p*-value
*Mycobacterium tuberculosis* (%)	49 (18.8)	35 (21.7)	11 (14.7)	0.20
*Candida species* (%)	39 (17.6)	23 (15.3)	13 (24.1)	0.18
*Klebsiella pneumonia* (%)	27 (12.2)	19 (12.7)	7 (13.0)	1.00
*Pseudomonas aeruginosa* (%)	16 (7.2)	12 (8.0)	2 (3.7)	0.35
*S*.*viridans/mitis* (%)	16 (7.2)	8 (5.3)	8 (14.8)	0.07
*Acinetobacter baumanni* (%)	11 (5.0)	7 (4.7)	3 (5.6)	1.00
*Pseudomonas alcaligenes* (%)	11 (5.0)	6 (4.0)	3 (5.6)	0.71
*Moraxella catarrhalis* (%)	10 (4.5)	7 (4.7)	1 (1.9)	0.45
*Escherichia coli* (%)	6 (2.7)	4 (2.7)	2 (3.7)	0.67
*Haemophilus influenza* (%)	4 (1.8)	2 (1.3)	2 (3.7)	0.31
*Staphylococcus aureus* (%)	4 (1.8)	3 (2.0)	1 (1.9)	1.00
*Streptococcus pneumoniae* (%)	4 (1.8)	4 (2.7)	0 (0)	0.58
*Enterobacter spp*. (%)	3 (1.4)	1 (0.6)	1 (1.9)	0.48
*Citrobacter* (%)	3 (1.4)	2 (1.3)	0 (0)	1.00
*Morganella morgani* (%)	2 (0.9)	2 (1.3)	0 (0)	1.00
*Pneumocystis jirovecii* (%)	0 (0)	0 (0)	0 (0)	-
Other (%)	8 (3.6)	6 (4.0)	1 (1.9)	0.67
Any bacteria (%)	121 (54.8)	83 (55.3)	30 (55.6)	0.98
Any pathogen(s) (%)	170 (59.4)	115 (62.8)	32 (41.0)	0.0007

In 40 patients multiple potentially pathogenic microorganisms were found. *Mycobacterium tuberculosis* was the most common co-infection and was present in 28 of the 40 patients. The most common co-occurrence was *Mycobacterium tuberculosis* and *Candida spp*. in 9 patients. Other co-occurrences included *Mycobacterium tuberculosis* and *Klebsiella pneumoniae* (found in five patients), *Mycobacterium tuberculosis* and *Pseudomonas aeruginosa* (found in three patients), and *Mycobacterium tuberculosis* and *Pseudomonas alcaligenes* (found in three patients).

Potential pathogens identified in sputum samples in patients with pneumonia. Percentages were calculated based on the total number of patients with a given type of culture sent (n = 260 for *M*. *tuberculosis* diagnostics and n = 221 for bacterial culture and *candida species* in the total cohort; n = 161 for *M*. *tuberculosis* diagnostics and n = 150 for bacterial culture and *candida species* in the HIV infected patients; n = 75 for *M*. *tuberculosis* diagnostics and n = 54 for bacterial culture and *candida species* in the HIV uninfected patients). For 83 of 209 HIV-infected patients, sputum samples were sent for analysis, and for HIV-uninfected patients this was the case in 78 of 87 patients. There were no statistically significant differences between isolated pathogens in HIV infected and HIV uninfected patients. Other isolates included *Serratia marcescens*, *Kocuria spp*., *Rothia mucilaginosa*, Neisseria gonorrhoea, Staphylococcus epidermidis, *Proteus mirabilis*, *Burkholderia pseudomallei*, and *Gamella sanguinis*. Note that the pathogens for HIV-infected and HIV uninfected patients combined do not match the total number of isolates, as some patients have missing HIV status.

*Streptococcus pneumoniae* was identified in four patients between the ages of 20 and 59 years. *Pseudomonas aeruginosa* was identified in 16 patients with 87% of them being between 20 and 59 years, 87% were HIV positive, of whom 92% were on ART. *Klebsiella pneumoniae* was the most frequently identified gram-negative organism. Of the patients with *Klebsiella pneumoniae*, 74% consumed alcohol and 73% were HIV infected. *Mycobacterium tuberculosis* was identified in 31% of the study population and 76% of these patients were HIV-positive with 79% receiving antiretroviral treatment at the time of presentation.

Twenty-one patients (6.4%) were managed as outpatients and the remainder were admitted to the hospital. Mortality 30-days after enrolment was assessed in 236 patients. Overall, 30-day mortality in this group was 30.1%. Table 3 shows 30-day mortality rates for several of the identified micro-organisms. Patients with TB had higher 30-day mortality than patients without TB (14 of 34 patients (41%) versus 37 of 152 patients (24%); *p* = 0.047). Six patients with rifampicin resistance were assessed for 30-day mortality, and 50% percent of these patients had died. Patients with *Candida* did not have significantly higher 30-day mortality compared to patients without *Candida* (13 of 27 patients (48%) versus 36 of 131 patients (27%); *p* = 0.059). Other micro-organisms were not significantly associated with 30-day mortality. In patients with TB, the additional identification of *Candida* from their sputum was not significantly associated with 30-day mortality (5 out of 7 compared to 6 out of 14, *p* = 0.221). Age at diagnosis and sex were not significantly associated with mortality at 30 days (median 38.0 years (IQR 25.0–51.0) versus 41.0 years (IQR 30.0–53.0), *p* = 0.20 (Mann-Whitney U test); 30% versus 30%, *p* = 0.96). Age more than 65 years was not associated with mortality at 30 days (*p* = 0.21). Systolic blood pressure less than 90 mmHg or diastolic blood pressure less or equal to 60mmHg was also not associated with mortality at 30 days (*p* = 0.24). Respiratory rate more than 30 breaths per minute and serum urea >19 mg/dL were associated with increased mortality at 30 days (38% versus 25%, *p* = 0.05; 70% versus 32%, p = 0.03, respectively). Previous history of TB or HIV was not associated with mortality at 30 days (34% versus 27%, *p* = 0.24; 31% versus 31%, *p* = 0.96, respectively).

**Table 3:** 30-day mortality stratified by an isolated microorganism.

Organism	30-day mortality (%)	*p*-value
*Mycobacterium tuberculosis*	14/34 (41)	0.047
Rifampicin-resistant tuberculosis	3/6 (50)	0.65
*Candida spp*.	13/27 (48)	0.059
*Klebsiella pneumoniae*	7/20 (35)	1.00
*Pseudomonas aeruginosa*	4/11 (36)	1.00
*Pseudomonas alcaligenes*	3/8 (38)	1.00
*Moraxella catarrhalis*	2/7 (29)	1.00
*Acinetobacter baumanni*	0/6 (0)	0.17
Any bacteria identified	27/85 (32)	0.83
Any (myco)bacteria identified	27/103 (26)	0.36

*P-values were derived from* comparisons to 30-day mortality in all other patients who were followed up after 30 days, and for whom sputum samples were sent for diagnostics (a total of 186 patients for *M*. *tuberculosis* diagnostics and 158 patients for bacterial culture and *candida species*; for 138 patients both were available and for 206 either one or both were available).

## Discussion

Using the study protocol, we successfully screened 337 patients with pneumonia at the Adult University Teaching Hospital in Lusaka, Zambia. A total of 327 patients were enrolled, of whom 286 had sputum samples analysed for microbiological causes of pneumonia. The diagnostic yield of 59% was similar to that in other studies, notwithstanding the diagnostic limitations, such as limited availability of radiographic imaging [9]. The patients had varying clinical presentations including cough, chest pain, fever, and breathlessness. In this cohort, 70% of the patients were infected with HIV, which increases the chances of pneumonia from various causes due to an immunosuppressive state [9, 16]. Notably, we did not find any significant differences between HIV-infected and HIV uninfected patients concerning the pathogens found in sputum samples.

The most common pathogen was *Mycobacterium tuberculosis*, found in 19.6% of the participants and it was associated with increased 30-day mortality. The observed prevalence was similar to that in a study by Mateyo *et al*. in severely immunosuppressed patients with HIV [9]. TB presenting as acute pneumonia is a known phenomenon in sub-Saharan African, where overall TB prevalence is influenced by the high burden of HIV [3, 7, 10, 17, 18]. Previous studies show rates of TB in non-HIV infected persons in sub-Saharan Africa as high as 35% [10]. Of the 49 *Mycobacterium tuberculosis* isolated, nine were rifampicin-resistant (18.4%). In this study, we could not establish whether they were multi-drug resistant, poly-resistant, or extensively drug-resistant strains.

*Candida* species were isolated in 17.5% of sputum samples and co-infection with *Mycobacterium tuberculosis* was seen in 23% of these. The synergistic growth of *Candida* has potential significance in pulmonary TB patients, but this is still a subject of investigation [19]. Thirty-day mortality in TB patients who also had *Candida* isolated from their sputum was not significantly higher compared to TB patients without *Candida* in this study. Importantly, candida pneumonia cannot be diagnosed by isolation of the organism in sputum, as this most likely represents colonization. Diagnosis of candidiasis requires a positive blood culture, a positive culture from a normally sterile site (other than urine, sinuses, or respiratory tract), or a histological biopsy showing invasive candida infection [20]. An association between culturing of *Candida spp*. from sputum and immunosuppression was not established in this study.

Gram-negative organisms were the most common bacterial isolates other than *Mycobacterium tuberculosis* and were altogether identified in about 30% of the isolates. Eighty-six percent of the study population had received antibiotics before enrolment which likely increases the proportion of gram-negative infections, as standard antibiotic regimens are directed mostly at gram-positive microorganisms [21]. Additionally, gram-negative organisms were the most common organisms associated with *M*. *tuberculosis* co-infection, similar to findings of a study by Iliyasu *et al*. in Nigeria [22]. The commonest gram-negative organism isolated was *Klebsiella pneumoniae*, with a frequency (12.1%) similar to that found by Mateyo *et al*. in HIV-infected patients [9]. A rare opportunistic organism called *Pseudomonas alcaligenes* was isolated in 4.9% of the patients, of which only 55% had HIV infection. *Streptococcus pneumoniae* was only isolated in 1.8% of the samples tested, in sharp contrast with previous studies where *S*. *pneumoniae* was the most common cause of CAP [4, 23]. This may be explained by the fact that the study setting is a national referral hospital and patients with organisms such as *S*. *pneumoniae* were likely adequately treated at lower-level facilities and not referred to tertiary-level care. Furthermore, a significant proportion of our cohort had a past medical history of TB, and any subsequent lung damage might predispose patients to infections with *P*. *aeruginosa* [24].

*Pneumocystis jirovecii* was not isolated in any of the samples collected despite 64 participants being significantly immunosuppressed (patients with an HIV infection and a CD4-count less than 200 cells/μl). *Pneumocystis* pneumonia usually occurs in the context of severe immunosuppression [25, 26]. The low prevalence of *Pneumocystis* jirovecii could be because cotrimoxazole prophylaxis was used by most patients that qualified according to national guidelines for the management of HIV [15]. Furthermore, this could be influenced by the diagnostic methods used, as patients did not routinely undergo broncho-alveolar lavage and beta-D glucan measurement, direct fluorescence antibody testing or molecular testing for *Pneumocystis jirovecii* was not performed. Bronchoalveolar lavage gives a much better diagnostic yield for *Pneumocystis jirovecii* than sputum samples [11, 25].

Overall, 30-day mortality was 30% and isolation of *Mycobacterium tuberculosis* was associated with the highest mortality rates. The mortality rate was higher than in a study done by Ramirez *et al*. in the United States of America where mortality 30 days after admission was 13% as well as in a study by Nyamande *et al*. where overall in-hospital mortality was 17% [10, 27]. The high mortality is likely explained by the study setting, as patients found at this tertiary-level hospital were mostly referred after failed treatment at lower health facilities. This may have increased their chances of exposure to multiple antibiotics thus increasing chances of antimicrobial resistance and consequently higher risk of mortality. According to Zambian treatment guidelines, the organisms presumed to cause pneumonia include *Streptococcus pneumoniae*, *Mycoplasma pneumoniae*, *Staphylococcus aureus*, and atypical organisms. The recommended empiric first-line antibiotics, therefore, are benzylpenicillin and amoxicillin [28]. However, findings from this study revealed more gram-negative organisms than gram-positive organisms. The recommended first-line therapy would therefore not adequately treat the patients in this setting. It could therefore be considered to use a second- or third generation cephalosporin as a first-line treatment in this setting for patients who are severely ill, while awaiting the results from culture and sensitivity testing.

This study had several limitations. Firstly, we did not perform molecular testing for viruses and other organisms, blood cultures, or antigen testing. Viruses are a major cause of CAP in developed countries, and in some studies are more common than bacteria [5, 29–32]. At present, very little is known about viral aetiologies of CAP in sub-Saharan Africa [3]. Secondly, we did not perform tests to establish fungal aetiologies. We did find a high prevalence of *Candida* species in sputum samples but were unable to further identify fungi such as *Aspergillus*. This would be quite interesting, as HIV infection is a known risk factor for fungal infections [3]. Third, we did not evaluate the quality of submitted sputum samples, which might have led to reporting of micro-organisms from suboptimal quality samples. Fourth, there were missing data on several data points, although this did not exceed 5% of the study population, except for culture availability, HIV status, and 30-day mortality. This introduces a degree of uncertainty, mainly with regard to the observed mortality, as it is well possible that the mortality rate was higher for patients that were lost to follow up. Finally, the study population is likely not representative for all Zambian patients with CAP, as UTH is a referral hospital and patients from lower-level facilities who improve under first-line treatment or who are not very ill are probably less likely to be referred. In addition, our study population is not representative of most other populations with CAP, as a large proportion of the cohort can be considered to be severely immunocompromised due to HIV. We did not find any significant differences in potential pathogens isolated between patients with and without HIV, but these comparisons are limited by a low sample size for any specific isolate.

## Conclusion

The study investigated the aetiology and prognosis among adult patients with CAP at the University Teaching Hospital in Lusaka, Zambia. HIV prevalence in the study population was 71% but was not associated with the isolation of different pathogens from sputum samples. *Mycobacterium tuberculosis* was the most common cause of CAP, found in one of five CAP patients. *Candida* species were commonly found in sputum samples (17.5%), but their significance was unknown. Gram-negative organisms were isolated in about 30% of the study population. Mortality after 30 days was 30% and infection with *M*. *tuberculosis* was associated with increased 30-day mortality. The causes of CAP that we identified in this setting differ from previous studies and would mostly not be covered by empiric antibiotics such as penicillins. This emphasizes the value of microbial diagnostics, as well as the need for locally informed treatment guidelines.

## Supporting information

S1 Data (XLSX)
